# Treatment of Varicose Veins

**Published:** 1847-01

**Authors:** F. C. Skey


*Treatment of Varicose Veins.* By F. C. Skey, Esq., F. R. S.—Mr. Skey observes that, the practice that he recommends in cases of varicose veins, has been successful in his hands for many years; it consists in the entire obliteration of the trunks and many of the branches of the saphena veins. both major and minor, by very small caustic issues, applied on the projecting veins, however large or numerous they may be; and in suggesting which, I believe I can pledge myself to two conditions; first, that the function of the vein on which the issue is made, is from that hour suspended; and second, that the operation of the issue so applied is unattended with danger of any kind. The operation appears to be complete, and immediate. The current of blood is arrested; nor is the obstruction to the circulation thus caused, apparent in any symptom of congestion resulting from it.

The material I employ for this purpose, is the Vienna paste, a compound of quick lime and caustic potash, in the proportion of five of the former to four of the latter. These ingredients are mixed into a paste with spirits of wine; and in these proportions which I cannot pronounce to be strictly those of the "Pate de Vienne," a caustic may be applied, which will readily destroy the surface without causing the excessive pain attending the application of the pure potash alone, nitric acid, or other similar agents. Under some circumstances it may be desirable to diminish the proportion of the caustic. The limb should be examined in the upright position of the body; the projecting veins marked with ink to the number requisite, including all the most salient points in the trunk and branches.

While in the horizontal posture the places thus marked, should be insulated with a circle of plaster of three or four thicknesses, the diameter of which will vary with that of the swelling beneath, but it is very desirable on all accounts to make it as small as possible. I have found one-quarter to one-third of an inch to suffice in the plaster, and the issue itself, will not exceed the size of a fourpenny piece or less.

The caustic may be removed at the expiration of half an hour, and the leg dressed and rolled lightly. In the course of a week or ten days the eschars come away, and the wounds are healed by ordinary means.—*London Med. Gaz.—South. Jour. Med.*

